# LEDGF/p75 has increased expression in blasts from chemotherapy-resistant human acute myelogenic leukemia patients and protects leukemia cells from apoptosis *in vitro*

**DOI:** 10.1186/1476-4598-6-31

**Published:** 2007-04-23

**Authors:** Tien-sheng Huang, Line M Myklebust, Endre Kjarland, Bjørn Tore Gjertsen, Frederic Pendino, Øystein Bruserud, Stein Ove Døskeland, Johan R Lillehaug

**Affiliations:** 1 Department of Molecular Biology, University of Bergen, Bergen, Norway; 2 Institute of Medicine, Hematology Section, Haukeland University Hospital, Bergen, Norway; 3 Department of Biomedicine, University of Bergen, Bergen, Norway; 4 Gades Institute and Department of Pathology, University of Bergen, Bergen, Norway

## Abstract

**Background:**

Relapse due to chemoresistant residual disease is a major cause of death in acute myelogenous leukemia (AML). The present study was undertaken to elucidate the molecular mechanisms of chemoresistance by comparing differential gene expression in blasts from patients with resistant relapsing AML and chemosensitive AML.

**Results:**

About 20 genes were identified as preferentially expressed in blasts pooled from patients with resistant disease, as compared to chemosensitive AML blasts, based on differential gene expression screening. Half of these genes encoded proteins related to protein translation, of these a novel protein related to the ribosomal stalk protein P0. Other upregulated mRNAs coded for cytochrome C oxidase III, the transcription factors ERF-2/TIS11d, and the p75 and p52 splice variants of Lens Epithelial Derived Growth Factor (LEDGF). Analysis of blasts from single patients disclosed that LEDGF/p75 was the most consistently upregulated mRNA in resistant AML. Transfection experiments demonstrated that LEDGF/p75 and p52b antagonized daunorubicin-induced and cAMP-induced apoptosis in an AML cell line. Also HEK-293 cells were protected against daunorubicin by LEDGF/p75 and p52b, whereas LEDGF/p52 splice variants lacking exon 6 had proapoptotic effects. Interestingly, full length LEDGF/p75 protected against truncated pro-apoptotic LEDGF/p75.

**Conclusion:**

Our results provide evidence for an association between the overexpression of genes encoding survival proteins like LEDGF/p75 and chemo-resistance in acute myelogenous leukemia. LEDGF/p75 has previously not been shown to protect against chemotherapy, and is a potential drug target in AML.

## Background

Acute myeloid leukemia (AML) is an aggressive malignant disorder characterized by neoplastic proliferation and accumulation of immature myeloid cells. Most patients achieve complete hematological remission after initial induction chemotherapy, but a large part of these patients will later develop leukemia relapse due to chemotherapy-resistant residual disease [1-3]. The overall long-term (5 years) AML-free survival is therefore less than 50% even for younger patients who receive the most intensive therapy [2,3]. Chemoresistance can manifest either as primary resistance to the induction therapy or as AML relapse following initial chemotherapy-induced hematological remission. A high frequency of AML relapse is especially observed in certain patient subsets characterized by persistent leukemic disease after the initial induction cycle or by high-risk cytogenetic abnormality [4,5]. Many of these abnormalities affect genes that encode proteins involved in the regulation of gene transcription [1]. Chemoresistance after treatment with anticancer agents may also be linked to perturbed gene expression [6,7]. These observations are consistent with altered gene expression being involved in chemotherapy resistance. This notion is further supported by two recent clinical studies describing association between long-term disease-free survival and particular gene expression profiles identified by cDNA microarray screening [8,9]. The aim of the present study was to identify genes overexpressed in chemoresistant AML and in native AML cells derived from patients with documented or high risk of clinical chemoresistance. For this purpose, we used differential hybridization of pooled RNA transcripts and confirmed the findings for each individual patient using RNA dot-blot and RT-PCR analysis. Our study demonstrated increased expression in chemoresistant cells of several gene products, notably lens epithelial-derived growth factor (LEDGF)/p75 and a novel gene called AML resistance associated protein (ARAP), similar to ribosomal protein P0. Enforced expression of the LEDGF/p75 and p52b genes protected against anthracycline-induced apoptosis. Interestingly, other less expressed splice variants of LEDGF were strong inducers of apoptosis, but their effect was overcome by co-expression of the full length variant. Our study suggests that AML cells express so far little known or unknown gene products able to protect against chemotherapy, and that naturally occurring pro-apoptotic splice variants can give clues to domains of these proteins that can be pharmacological therapy targets.

## Results

### Identification of genes overexpressed in AML relapse cells

More than 25,000 colonies of our leukemia cDNA library were screened with cDNA probes made from pooled leukemia cell RNA from patients with chemosensitive (L1-3) AML and from patients with AML resistant/relapsed leukemia (R1–R5). We identified about 120 colonies with increased hybridization to cDNA probes from R-cells. They were subjected to a secondary screening, in which 19 putative genes were found to be upregulated more than 2-fold. The genes were identified by DNA sequencing (Table 1). One of the genes with increased (5-fold) expression in the R-cells has not been described earlier and consequently has not been associated with any function so far. We refer to its predicted product as AML resistance associated protein (ARAP). Two other genes coded for known growth- and survival-associated transcription factors, i.e. lens epithelium-derived growth factor (LEDGF) and the EGF response factor 2 (ERF-2). Most of the upregulated genes coded for ribosomal proteins. The translation-related Elongation Factor (EF)-1α also showed increased expression. Three upregulated genes coded for mitochondrial proteins, and two others for proteins involved in antigen presentation or protein degradation. We also noted an upregulation of lysozyme.

**Table 1:** Genes preferentially expressed on chemotherapy-resistant AML relapse

Genes identified	Primary differential screening mRNA expression	Individual patients means of mRNA expression	
	R/L	R/L	H/L
Novel			
ARAP	5.0	40.	0.9
			
Transcription factors			
LEDGF/p75	4.8	7.7	2.8
ERF-2/TS11d	2.2	3.3	1.3
			
Translation-related			
EF-1	3.2	3.2	3.4
rpL3	2.9		
rpL6	4.5	2.8	4.7
rpL11	2.7		
rpL34	2.7		
rpL41	3.6		
rpS4	5.3	3.8	3.5
rpS12	3.2		
rpS24	2.7		
rpS25	2.7		
			
Mitochondria-related			
CytOX II	2.4	2.1	2.5
CytOX III	4.0	2.3	3.7
ND4	2.7		
			
Others			
Lysozyme	4.2	2.3	4.1
MHC-DRA	3.6		
PSME1/pAI	2.4		

The results are based on primary differential screening of an AML cDNA library of more than 25,000 colonies. Probing was with cDNa probes derived from RNA of pooled native AML blasts from either patients sensitive to chemotherapy or from patients relapsing after several chemotherapy treatment courses. It also shows the means of mRNA expression in relapsed AML blasts relative to sensitive blasts from individual patients. [32P] cDNA derived from individual AML patient blasts (L1-3, R1-5 and H1-5) were used as probes for dot-blot analysis against nine of the genes found up-regulated in pooled RNA from resistant AML blasts. Only genes with expression ratio exceeding 2 are presented. L: low risk blasts; H, high risk blasts; ARAP, AML resistance associated protein; rp, ribosomal protein; CytOX II, III, cytochrome C oxidase subunit II and III; ND4, NADH dehydrogenase subunit 4; EF-1, elongation factor 1; PSME1/pAI, proteasome activator 28 subunit I; ERF-2/TIS11d, EGF response factor-2/human homolog of TIS11d; LEDGF, lens epithelium derived growth factor, and MHC-DRA, major histocompatibility complex class II DR alpha.

**Figure 1:**
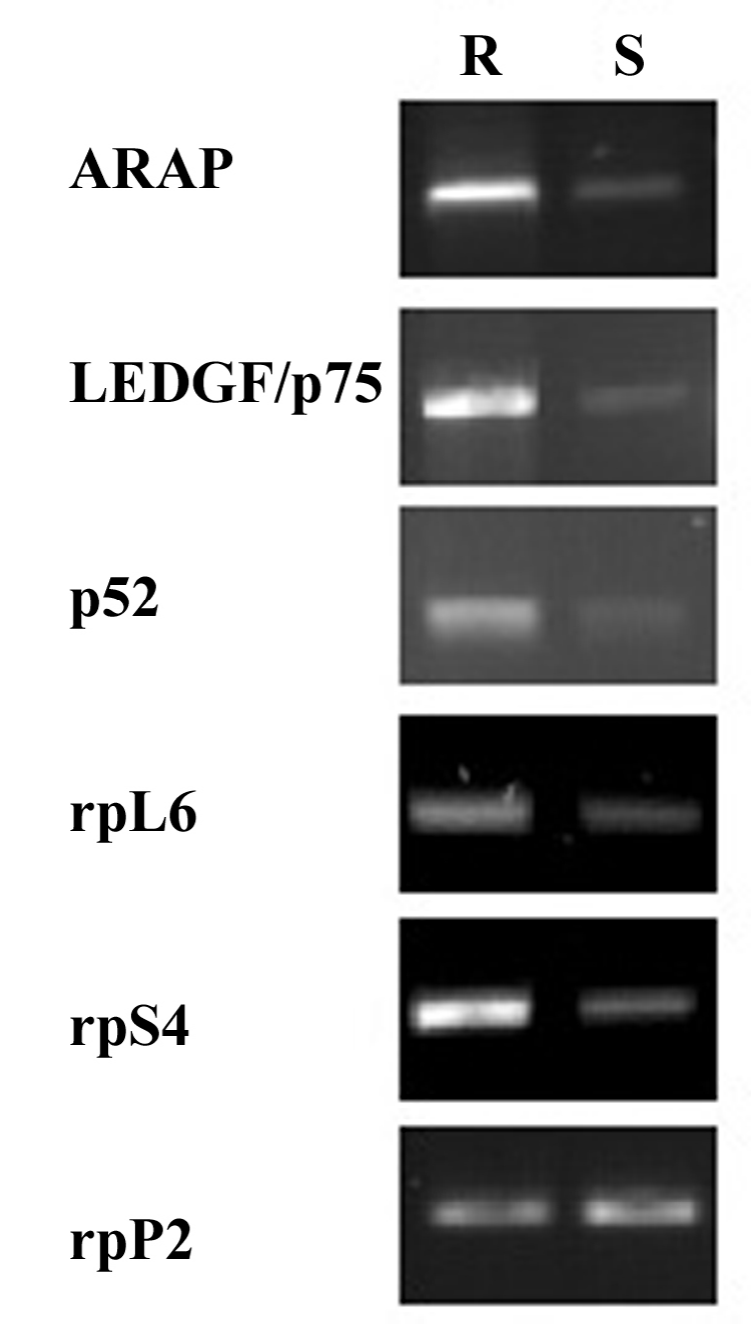
**Confirmation of preferential gene expression in chemotherapy-resistant AML**. The expression of the genes found by primary differential screening to have the highest relative expression in resistant AML blasts (Table 1) was reinvestigated by RT-PCR of RNA pooled from resistant (R; left column) or low risk (L; right hand column) AML blasts. The level of rpP2, used to normalize expression of the other genes, was also studied. The R-blasts were from patients with relapse from chemotherapy (R1-5) and the L-blasts from chemo-responsive patients (L1-3). See the Material and methods section for further details on patients, cells and RT-PCR procedure.

The screening result for the genes with highest relative expression in R-cells was validated by RT-PCR in the pooled cDNA probes prepared from RNA of L- and R-cells. Two sets of specific primers were used and rpP2 served as a control for equal loading. The novel gene ARAP was confirmed to be relatively overexpressed in the R-cells (Fig. 1). The LEDGF/p75 gene generates two transcripts by alternative RNA splicing, LEDGF/p75 and p52 mRNAs [10-12]. Both these transcripts showed increased levels in relapsed/chemoresistant AML, as did the transcripts for the ribosomal proteins L6 and S4 (Fig. 1). From these data, we conclude that the R-cells had increased expression of several genes associated with transcription, translation, mitochondrial function, and protein processing, suggesting a possible reprogramming of several pivotal cell functions of the blast cells from patients with chemoresistant AML.

We have recently found profound differences in phosphoprotein signaling cascades between AML cells from patients with low-risk and high-risk of relapse [13]. Our L-cells were from low-risk patients, and their expression of 9 of the genes presented in Table 1 was studied in cells from individual patients. For comparison, we studied the expression of the same genes in cells from individual patients with high risk (H-cells) of relapse and the cells from patients with resistance (R-cells) to chemotherapy regimens (Table 1). It appeared that ARAP expression was enhanced in 4 of the 5 R-cell patients, average more than 4-fold, but not increased in the H-cells. High expression of this gene could therefore be a consequence of the chemotherapy. The genes encoding the transcription factors LEDGF/p75 and ERF-2 were also generally more upregulated in the R-cells than in the H-cells (Table 1). The other genes studied were upregulated in at least half of the patients whose R- and H-cells were studied. They were generally equally or more upregulated in the H-cells than in the R-cells, suggesting that previous chemotherapy was not required for the overexpression. We conclude that high-risk and low-risk AML cells differ not only with respect to phospho-signalomics [13], but also with respect to expression of the genes associated with AML relapse. Most R and H patients had increased expression of a number of the resistance-associated genes. We focused in more detail on the LEDGF/p75 because it had the highest relative expression in the R-cells relative to the L-cells among the differentially expressed genes tested (Table 1) and earlier studies indicating LEDGF/p75 to be involved in cell survival [14,15].

### LEDGF/p75/p52 mRNA splice variants in human AML cells

**Figure 2:**
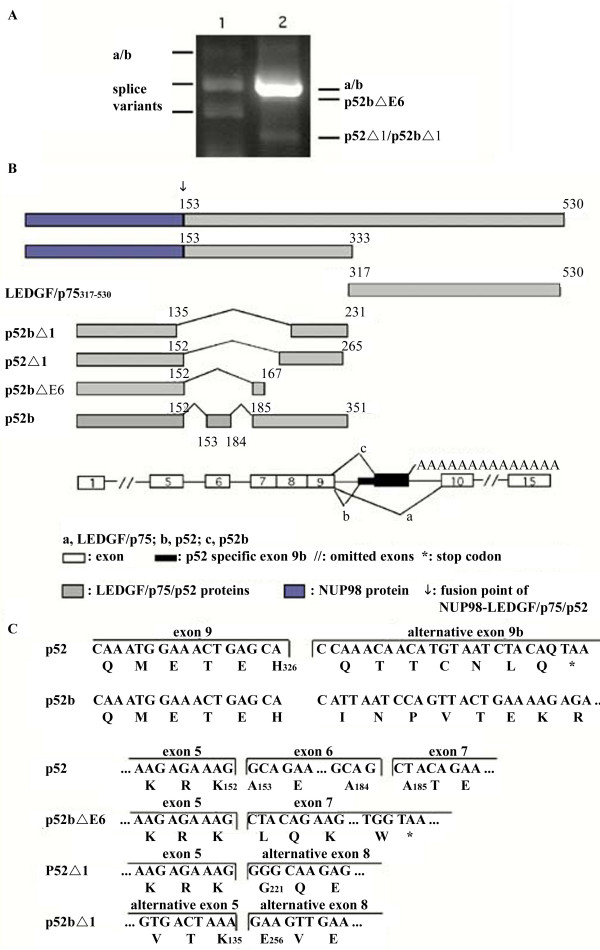
**The expression and characterization of LEDGF/p75 splice variants in NB4 cells**. A) The splicing pattern of LEDGF/p75 mRNA in NB4 cells as revealed by RT-PCR. The primers (p75-1; from exons 1 and 9) used for lane 1 (left) and (p75-2; exons 3 and 8) used for lane 2 (right) were from the common region of LEDGF/p75 and p52. The identity of the splice variants marked in lane 2 was verified by DNA sequencing. B) Schematic representation of the p52 splice variants. The identical N-terminal domain of LEDGF/p75 and p52 is indicated as exon 1 to exon 9. The p52 variant contains an alternative exon 9b with a poly (A) tail at its C-terminal end, whereas C) p52b differs from p52 by lacking 55 bases in the alternative exon 9b. The low expression p52bΔE6 variant lacks exon 6 at its N-terminal region and has a frameshift that introduces several stop codons, whereas p52Δ1 and p52bΔ1 lack exons 6, 7 and parts of 5 and 8. // indicates omitted exons in the figure.

Both LEDGF/p75 and p52 were associated with chemoresistance in native human AML cells (Fig. 1). In subsequent experiments, the full-length LEDGF/p75 and p52 cDNAs were cloned by RT-PCR from the promyelocytic leukemia NB4 cells. Additional novel spliced LEDGF/p75 mRNA transcripts were detected by RT-PCR of the overlapping region between LEDGF/p75 and p52, and these splicing patterns were confirmed by RT-PCR using primers derived from exon 3 and exon 8 (Fig. 2A). Subcloning and sequencing of p52 cDNAs derived from NB4 AML cells identified four previously unknown splice variants of the LEDGF/p75/p52 gene (summarized in Fig. 2B). One, p52b, had a p52-specific exon 9b. The putative amino acid sequence of this novel and well expressed (Fig. 2A) p52 mRNA variant was identical to p52 except for 25 extra amino acid residues at the C-terminal end due to an altered open reading frame. Three different low expression (Fig. 2A) variants without exon 6 are referred to as p52bΔE6 (Fig. 2B), p52Δ1, and p52bΔ1. The P52bΔE6 variant coded for a putative truncated protein due to a stop codon introduced into the P52b reading frame, whereas p52Δ1 and p52bΔ1 encoded proteins with gross deletions (lack of exons 6, 7 and large parts of 5 and 8).

LEDGF/p75 can be expressed in a number of splice variants in AML cells. We wanted to know the possible functional significance of the overexpression of LEDGF/p75 in AML cells from patients with chemoresistance, and the significance of the splice variants of p52 described. For this purpose, we first used Flp-In 293 cells transfected with the various genes and analyzed for induction of spontaneous apoptosis or for ability to modulate the apoptosis induction by daunorubicin.

### Overexpression of LEDGF/p75 protected against daunorubicin-induced apoptosis, whereas LEDGF/p52 splice variants without exon 6 were pro-apoptotic

**Figure 3:**
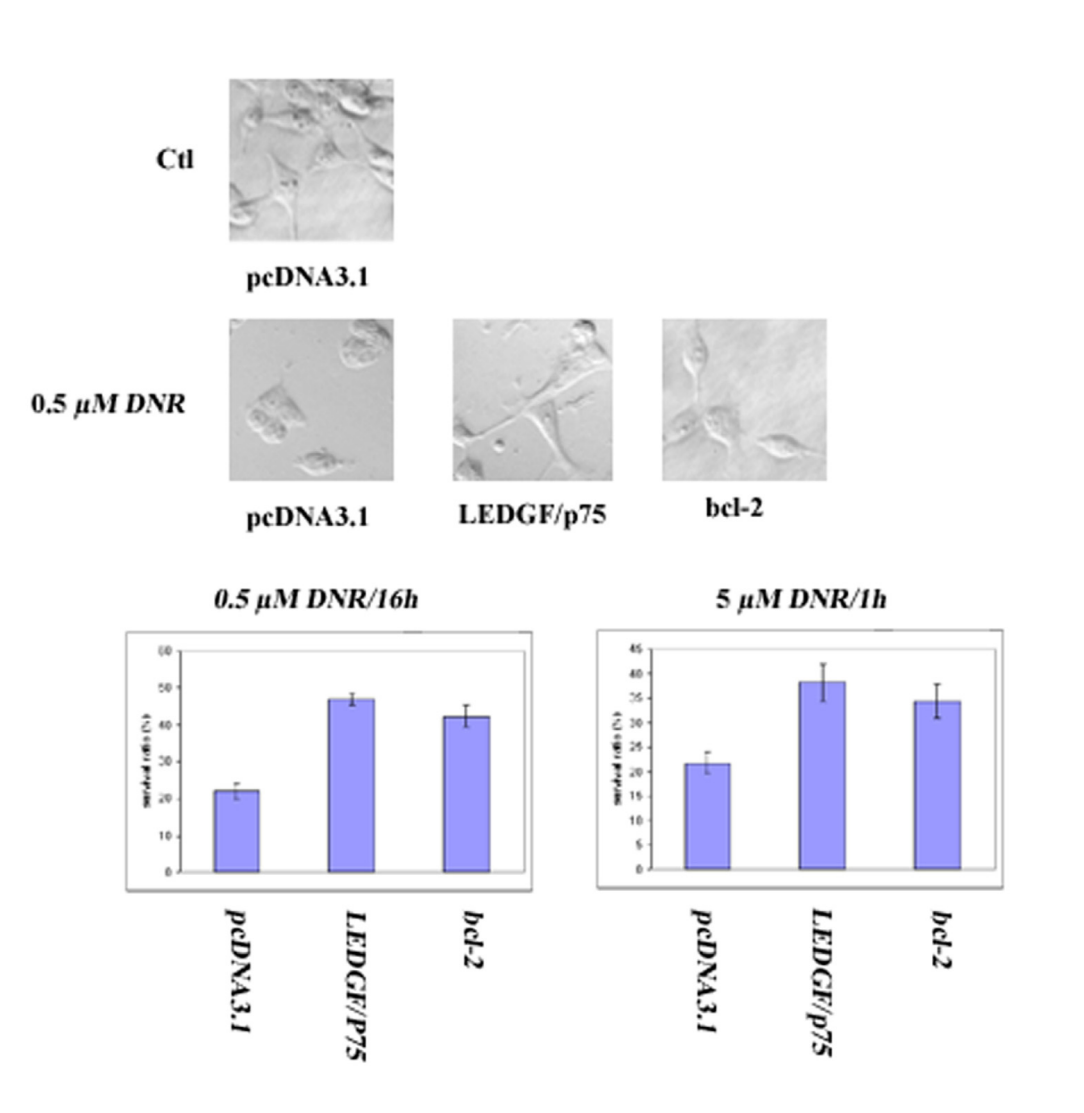
**Effects of LEDGF/p75 and bcl-2 on daunorubicin-induced apoptosis**. Flp-In 293 cells were transfected with control vector pcDNA3.1 or vectors containing either LEDGF/p75 or bcl-2. All transfected cells treated with vehicle alone (Ctl) had normal morphology. After treatment with 0.5 μM daunorubicin (DNR) for 16 h only 22% of the cells transfected with control vector appeared morphologically viable, against 47 and 42% for cells transfected with either LEDGF/p75 or bcl-2. Also cells treated with 5 μM daunorubicin for 1 h were protected by transfection with either LEDGF/p75 or bcl-2. Cell viability was analysed as described in the Materials and Methods section. Results are presented as the mean of viable cells ± SEM of separate experiments performed in triplicate (n = 3).

We previously used transiently transfected HEK293 cells to show that Bcl-2 protects against daunorubicin-induced, but not okadaic acid induced apoptosis [16]. In the present study, we compared the ability of transfected LEDGF/p75 and Bcl-2 to protect against daunorubicin induced apoptosis, using either a moderate drug concentration (0.5 μM) for an extended period of time (16 h) or a high concentration (5 μM) for a short period (1 h). In either case, the protective effect of LEDGF/p75 was comparable with that of Bcl-2, transfected under similar conditions (Fig. 3). We found significantly increased survival, as compared to cells transfected with empty vector, for cells transfected with LEDGF/p75 (p < 0.01). We conclude that LEDGF7p75 consistently overexpressed in resistant and high-risk AML patient cells were indeed able to protect against a first line anti-leukemic drug.

**Figure 4:**
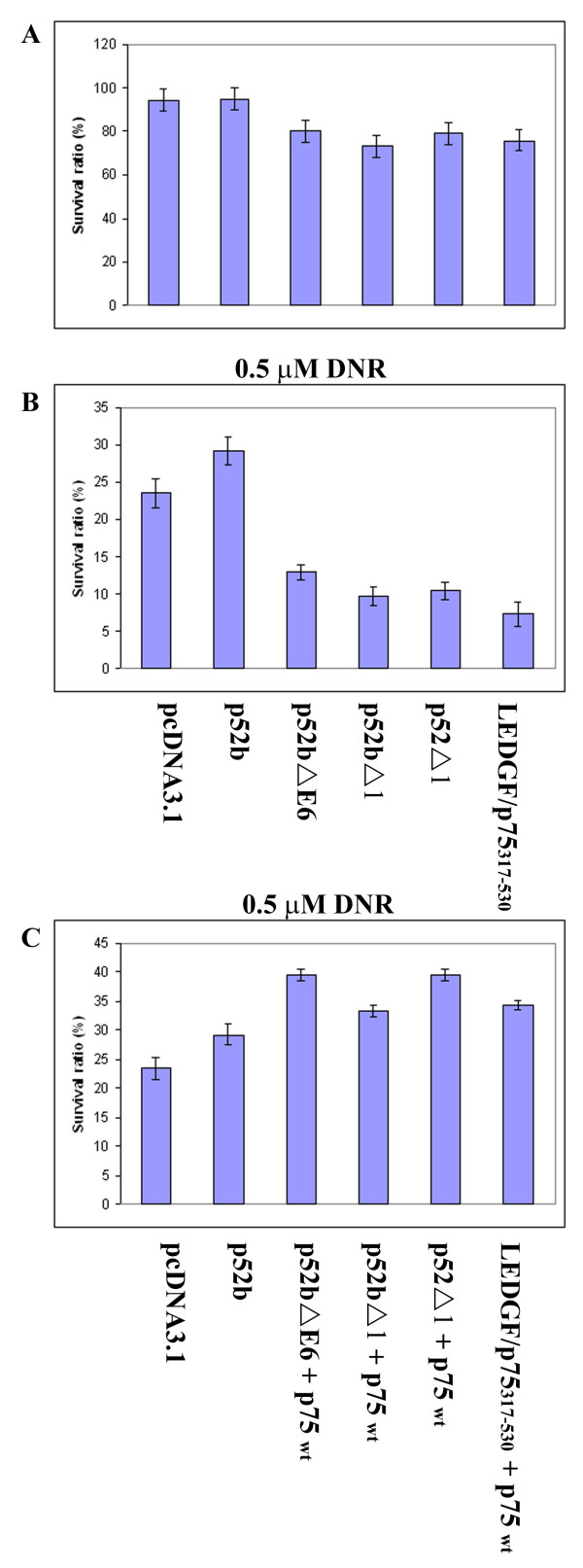
**Effects of LEDGF/p52 splice variants on spontaneous and daunorubicin-induced apoptosis**. Flp-In 293 cells were cultured in medium without (A), or with 0.5 μM daunorubicin (DNR) for 16 hours (B, C). The cells were transfected with control vector (pcDNA3.1), p52b (with exon 6), with p52Δ1, p52bΔ1 or p52bΔE6 (all of which lack exon 6), or with LEDGF/p75317-530, i.e. the part of the LEDGF/p75 gene (exons 10–15) that do not overlap with p52. In panel C, cells were co-transfected with full length LEDGF/p75 and p52Δ1, p52bΔ1, p52bΔE6, or C-LEDGF/p75317-530. Scoring of cell viability and other experimental conditions are as described in the legend to Fig. 5.

We investigated next the effect of LEDGF/p75 splice variants on *in vitro *apoptosis of Flp-In 293 cells. First, the effect of the splice variants was investigated for spontaneous apoptosis in cells cultured in medium alone. A decreased number of viable cells was observed for cells transfected with the three splice variants lacking exon 6 (p52Δ1, p52bΔ1, p52bΔE6, see Fig. 2) compared with cells transfected with the control vector (Fig. 4). A similar decrease in viability was also observed when testing an artificial LEDGF/p75_317–530 _gene segment encoding only the 317–530 amino acids of the C-terminal domain and thereby lacking overlap with p52 (Fig. 4). In contrast, a high viability was observed both when testing cells transfected with the control vector and with the p52b splice variant containing exon 6. The non-viable cells had morphology consistent with apoptosis in all these cultures. Thus, the 3 splice variants without exon 6 seemed to mediate proapoptotic effects. Secondly, a similar difference between splice variants without exon 6 and the p52b variant was observed for daunorubicin-induced apoptosis (0.5 μM daunorubicin, 16 hrs exposure), but the differences were smaller than for spontaneous apoptosis (Data not shown). Finally, the effect of the four p52 splice variants was also determined for Flp-In 293 cells transfected with LEDGF/p75 and exposed to daunorubicin as described above. LEDGF/p75 antagonizes daunorubicin-induced apoptosis, and the number of viable cells did not differ between the four p52 splice variants in these experiments.

### Overexpression of LEDGF/p75 and p52b in IPC81 AML cells protected against apoptosis induced by daunorubicin or cAMP analog

**Figure 5:**
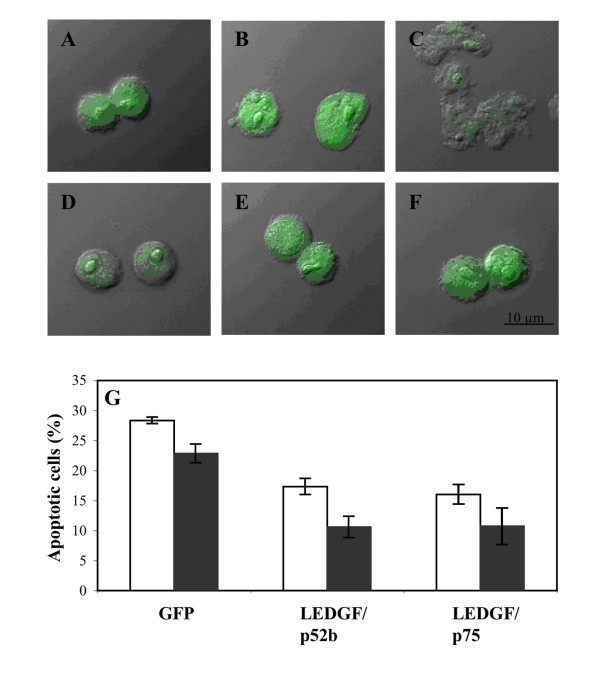
**Enforced expression of LEDGF/p52b and LEDGF/p75 counteracted leukemic cell death induced by DNR or cAMP analog**. The upper panels show differential interference contrast micrographs of IPC-81 leukemia cells with enforced expression of GFP (A-C) or bicistronic expression of LEDGF/p75 and GFP (D-F), and treated 20 h with vehicle (A, D), 10 nM DNR (B, E), or 10 μM 8-pCPT-cAMP (C, F). The lower panel (G) shows % apoptosis (mean ± SEM, n = 5) of IPC cells after 20 h treatment with 10 nM DNR (open bars) or 10 μM 8-pCPT-cAMP (solid bars). Note the lower % apoptosis in cells expressing LEDGF/p75 and LEDGF/p52b than in cells expressing GFP.

Since the LEDGF/p75 overexpression was found in resistant AML cells, it was important to know whether overexpression of LEDGFp75 and p52b also could protect AML cells. For this, we chose the highly anthracyclin-sensitive IPC-81 rat AML cell line [17], which originates from the BNML rat model of leukemia, known to be a reliable predictor of treatment success for chemotherapy agents in current clinical use against AML [18]. These cells are also highly sensitive to apoptosis induction by activation of the cAMP-dependent protein kinase [19,20]. In order to achieve efficient gene transfer, the LEDGF/p75 and p52b constructs were delivered via a retroviral system. In order to visualize transduced cells, the LEDGF/p75 genes were cloned in tandem with a GFP gene governed by an internal ribosome entry site. We found that cells with enforced expression of either LEDGF/p75 or p52b were significantly protected against apoptosis induced by either daunorubicin or cAMP analog (Fig. 5). We conclude that both LEDGF/p75 and p52b are able to protect against AML death *in vitro*, suggesting that their consistent overexpression in AML cells from treatment – resistant AML is not coincidental.

### Effects of daunorubicin on LEDGF/p75 expression in human NB4 AML cells

**Figure 6:**
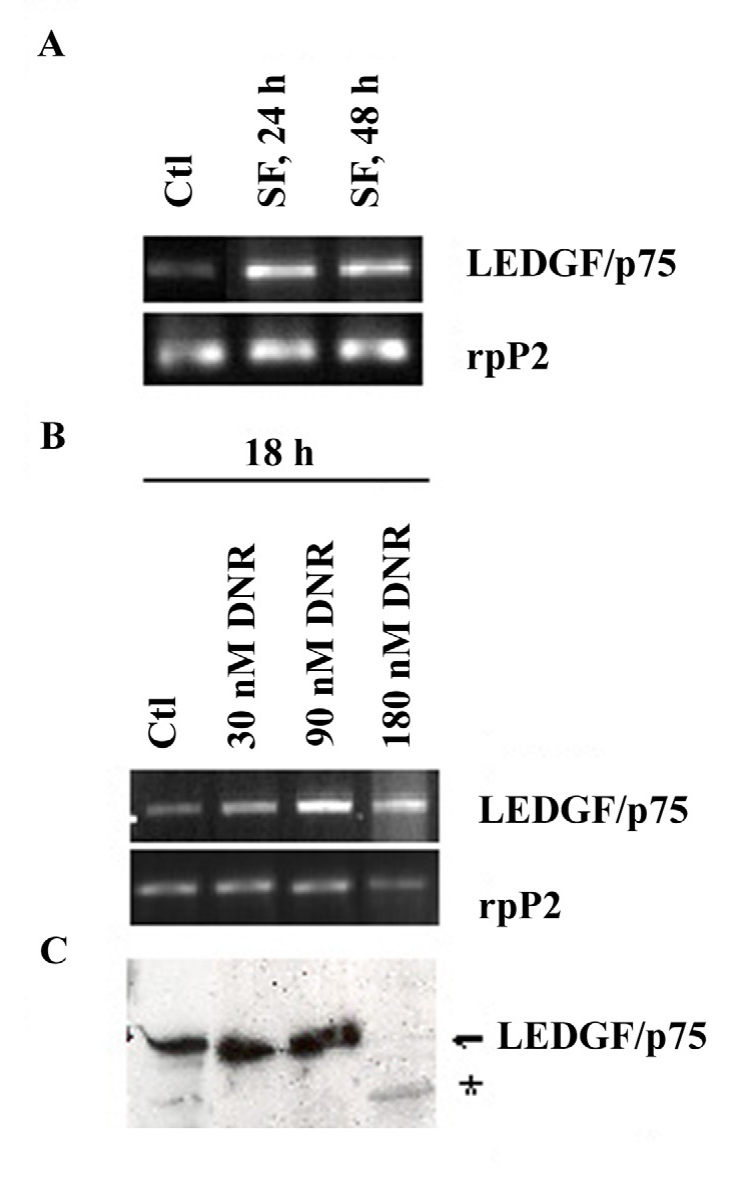
**Expression of LEDGF/p75 mRNA and protein in NB4 cells treated with daunorubicin**. A) Expression of LEDGF/p75 mRNA in NB4 cells treated with serum starvation analyzed by RT-PCR. Cell were cultured under serum-free (SF) conditions for 24 and 48 hours before analysis; untreated NB4 cells served as control cells (Ctl). Primers for the rpP2 gene were also included as a control. The RT-PCR results are representative of at least three independent experiments. B) The expression of LEDGF/p75 and ARAP mRNA in NB4 cells exposed for 18 h to daunorubicin (DNR) at 30, 90, or 180 nM. C) Western blot analysis of NB4 cells exposed to DNR as described above. Note the disappearance of full length LEDGF/p75 (75 kDa) and formation of a degradation product * (58 kDa) after exposure to 180 nM DNR.

The regulation of LEDGF/p75 expression was investigated in NB4 human AML cells. We analyzed LEDGF/p75 mRNA expression in serum-starved NB4 cells, and significantly increased LEDGF/p75 mRNA levels were observed after 24 and 48 hours (Fig. 6A). We then investigated the effect of daunorubicin on expression of LEDGF/p75 mRNA and its protein. rpP2 cDNA as a standard reference in the RNA preparations. LEDGF/p75 mRNA was significantly increased in cells treated with 30 nM and 90 nM daunorubicin for 18 hours (<10% and 40–50% apoptosis, respectively), whereas LEDGF/p75 mRNA levels were reduced and a majority of cells were apoptotic (approx. 90%) when cells were exposed to 180 nM daunorubicin (Fig. 6B). The levels of LEDGF/p75 protein in total protein extracts from NB4 cells were further analyzed by western blot analysis using anti-LEDGF/p75 antibodies. A 75 kDa band specific of LEDGF/p75 increased in the presence of 30 and 90 nM daunorubicin (Fig. 6C), whereas after exposure to 180 nM daunorubicin, degradation of LEDGF/p75 was observed with the formation of a novel protein band of approximately 58 kDa. This new protein seems to be a relatively stable LEDGF/p75 degradation product generated by caspase 3 mediated cleavage during apoptosis [21].

## Discussion

The present differential gene display analysis identified a number of genes with increased expression in relapsed AML. The increase of each mRNA varied between patients, in keeping with our recent study [13] which revealed different response of signaling pathways coupled to translational control in a similar panel of patient AML cells. Also array-based AML gene expression profiling studies [9,22-24] underscore the heterogeneity of AML and the need for personalized therapy [25,26].

A strikingly high proportion of the genes with increased expression in relapsed AML (Fig. 1) were related to protein translation. One of them, termed ARAP, has sequence similarities to ribosomal protein P0 and has not previously been cloned. The elucidation of ARAP function and the role proteins involved in protein translation will be the subject of future studies.

Importantly, we found increased mRNA levels in relapsed AML for the general transcriptional co-activator lens epithelium derived growth factor (LEDGF)/p75 and its splice product p52 [11]. The LEDGF/p75 mRNA was the most consistently upregulated in our series of relapsed AML. AML cells transfected with LEDGF/p75 or p52b were significantly more resistant to apoptosis induction *in vitro *by either daunorubicin or the presumed physiological apoptogen cAMP (Fig. 5). Detailed studies in HEK293 cells showed that overexpression of LEDGF/p75 protected against daunorubicin-induced death to a similar extent as bcl-2 (Figs. 3, 4), suggesting that increased LEDGF/p75 might enhance chemotherapy resistance also in non-AML cells. This finding is consistent with LEDGF/p75 protecting cells against stress such as serum deprivation [14]. LEDGF/p75 and p52 share the same 325 N-terminal residues, in which is found a PWWP domain (residues 1–100) interacting with stress response elements, a nuclear localization signal (residues 146 – 156), and an AT-hook motif (residues 179–198) [14]. Although p52 only has 8 unique C-terminal amino acids, it can interact selectively with the splicing factor ASF/SF2 [12]. The unique C-terminal 205 residues of LEDGF/p75 harbor a hepatoma derived growth factor homology domain that binds to chromatin even in the absence of a functional NLS [27]. The pro-survival function of LEDGF/p75 is believed to depend on the 44 C-terminal residues since LEDGF/p75 (1–486) failed to protect hepatoma cells against serum deprivation [21]. We found that p52b, which lacks the C-terminal residues of LEDGF/p75 (Fig. 2) did not induce apoptosis but our data suggest that it has some protective activity against daunorubicin-induced death (Fig. 4). This suggests that the C-terminal 205 residues of LEDGF/p75 were dispensable for protection against cell death. In fact, enforced expression of LEDGF/p75 (317–530) not only failed to protect against daunorubicin but induced significant death by itself (Fig. 4). The finding that p52 is not pro-apoptotic helps understand why patient leukemic cells with cytogenetic abnormality, t(9;11), involving the NUP98 gene and encoding NUP98-LEDGF/p75/p52 fusion proteins, have a high expression of both the LEDGF/p75 and p52 fusion products [28-30].

LEDGF/p75 and p52 were also present in NB4 AML cells, in which we detected four additional novel p52 variants, three of which had deleted exon 6. One variant (p52bΔE6) lacked only exon 6, while the others (p52Δ1, p52bΔ1) also lacked exon 7 and parts of exons 5 and 8 (Fig. 2). Exon 6 codes for residues important both for the NLS and the AT-hook of LEDGF/p75, and its absence will therefore presumably abolish or alter the nuclear localization of p52. All three p52 splice variants lacking exon 6 enhanced rather than protected against daunorubicin- induced death (Fig. 4). This means that the LEDGF/p75 gene can create both pro- and anti-apoptotic proteins. We found that full-length LEDGF/p75 protected against all three pro-apoptotic splice variants of p52 as well as against the pro-apoptotic p75 (317–530) fragment of LEDGF/p75 (Fig. 4). This suggests that increased expression of full-length LEDGF/p75 may protect against pro-apoptotic p52 splice variants as well as against truncated LEDGF/p75 variants produced by caspase-dependent processing of LEDGF/p75 [21]. The high expression of LEDGF/p75 in relapsed AML may therefore protect against apoptosis both by stimulating the transcription of other survival genes and by partially blocking the effect of pro-apoptotic shorter versions of LEDGF/p75 and p52.

## Conclusion

In summary, relapsed as well as "high risk" AML cells have increased expression of several genes encoding novel as well as known anti-apoptotic proteins. Few of these genes have previously been implicated in AML drug resistance. The LEDGF/p75 gene, as well as several pro-apoptotic splice variants of p52, in NB4 and AML (IPC81) cells have not been described before. A striking observation is the heterogeneity with regard to which genes that are upregulated, identifiable in the present study. This confirms that AML is a heterogeneous disease. We consider LEDGF/p75 to be a potential, novel therapeutic target in relapsed AML since it was among the genes most consistently upregulated and because it provided resistance towards daunorubicin after transfection. The LEDGF/p75/p52 molecule has both pro- and anti-apoptotic subdomains, the exact extent and function of which is only beginning to be unraveled. One strategy for improved chemotherapy of cancer cells with overexpression of LEDGF/p75 can be to remove the anti-apoptotic function inherent in the residues coded by exon 6, by first studying the effect of abolishing the NLS and the AT-hook present in this region.

## Methods

### Patients

AML blasts were collected from the peripheral blood of 13 patients. Blasts were collected from 3 patients with chemo-sensitive AML at the time of diagnosis ("low risk", L1-3). All had complete hematological remission after the first induction cycle. Two are still alive 93 and 109 months after diagnosis, respectively; the third had 44 months of disease-free survival even though consolidation therapy could not be completed due to serious side effects. All three had low/intermediate risk of relapse [4,31] judged from cytogenetics (two inv(16), one normal) and no genetic Flt3 abnormality. AML cells were collected from five patients who had undergone previous intensive therapy, (i) two patients had relapse after intensive chemotherapy and allotransplantation, respectively, and (ii) three patients had primary resistant disease and did not reach remission after three induction cycles. For comparison, cells were collected at the time of primary diagnosis from patients with high risk of resistance/relapse [31-33]. AML ("high-risk", H1-5). Two patients (H1, 2) had AML secondary to primary myelodysplastic syndrome (MDS), and treatment was not attempted, whereas the three last patients (H3-5) later showed resistance to at least two different induction regimens. All human samples were collected in accordance with the Helsinki Declaration.

### Preparation of native AML blasts

Leukemic peripheral blood mononuclear cells (PBMC) were collected from peripheral blood, and isolated by density gradient separation (Ficoll-Hypaque; NyCoMed, Oslo, Norway; specific density 1.077) [34,35]. Cells were stored in liquid nitrogen until use. The percentage of blasts among leukemic PBMC exceeded 95% for all patients, as judged by light microscopy of May-Grünwald-Giemsa stained smears.

### Culturing, transfection and apoptosis scoring in cell lines

Flp-In 293 cells (Invitrogen) were cultured in DMEM medium supplemented with 10% heat-inactivated fetal bovine serum. The p52 specific splice variants were cloned into pcDNA3.1/V5-His TOPO (Invitrogen). LEDGF/p75 in pcDNA3.1/His was a generous gift from C. A. Casiano (St. Loma Linda University, USA). Cells were cultured and transfected using FuGENE-6 (Boehringer Mannheim) according to the manufacturer's protocol. After 24 h, cells were diluted to desired number and seeded into collagen coated 12-well plates. Cells were fixed by 4% buffered paraformaldehyde after DNR treatments. Cells were examined for daunorubicin-induced apoptosis by fluorescence microscopy after DNA staining [16,34]. For estimation of statistical significance of values for percentage of apoptosis, we used the non-parametric Wilcoxon paired comparison test.

### Cell culturing, virus production and assessment of apoptosis

The promyelocytic leukemia cell line IPC-81 and NB4 cells were generous gifts from Dr M Lanotte (INSERM U-496, Centre G Hayem, Hospital St Louis, Paris, France). The cells were cultured in Dulbecco's modified Eagle's medium (DMEM, Invitrogen) under standard conditions supplemented with 10% heat-inactivated horse serum (Invitrogen), streptomycin (5 μg/ml) and penicillin (5 U/ml). Cells were kept in logarithmic growth, and as a standard procedure, cell density was adjusted to 0.2 × 10^6 ^cells/ml before addition of drugs. The cyclic AMP analog 8-chlorophenylthio-cAMP (8-CPT-cAMP) was purchased from Biolog Life Science, Bremen, Germany. Daunorubicin (DNR) was purchased from Sigma-Aldrich Inc. (St Louis, MO, USA).

Phoenix-Eco virus producer cells and CRU5-IRES-GFP retrovirus vector were kindly provided by Dr. J. Lorens (Department of Biomedicine, University of Bergen) and the cells were maintained in DMEM supplemented with 10% heat-inactivated fetal calf serum (Invitrogen). The full-length cDNAs of LEDGF/p75 and p52b with Bst XI linker were cloned into CRU5-IRES-GFP.

Retrovirus were pseudotyped with the vesicular stomatitis virus glycoprotein (VSVG) and concentrated by ultracentrifugation essentially as described [36]. Briefly, 1.5 × 10^6 ^Phoenix cells plated the day prior to transfection using the calcium phosphate method, 2 μg retroviral plasmid and 2 μg VSV-G. After 48 hours, filtered supernatants were concentrated by centrifugation for 3 hours at 50 000 g, 4°C. IPC-81 cells (0.5 × 10^6^) were transduced by spin infection in which a mixture of virus and cells was centrifuged at 1200 g for 90 minutes at room temperature in the presence of protamine sulphate (5 mg/ml, Sigma-Aldrich). Routinely, 90–95% of the cells showed fluorescence 48 hours after infection. After treatment, cells were fixed in phosphate-buffered saline containing 2% formaldehyde. Screening for apoptosis was done blindly by two independent experienced evaluators using phase contrast microscopy to study surface morphology and nuclear morphology as visualised by the DNA-specific stain bisbenzimide, Hoechst 33342 (Sigma-Aldrich). Three different fields were randomly selected for counting at least 300 cells. All experiments were repeated at least five times. Percent apoptosis is defined as the ratio between cells with both membrane budding and chromatin condensation and the total of cells counted.

### Differential screening of a leukemia cDNA library

The construction of a leukemia cDNA library and hybridization was as described previously [37]. The SMART system (Clontech) was used to construct a cDNA library from the pooled RNA collected from AML patient cells (Table 1; S1 to S3 and R1 to R5). The UV-cross linked replica membranes with total over 25,000 colonies were subjected to primary screening by radio-labeled first strand cDNA from the pooled RNA of either "low risk" (L1-3) or "resistant" (R1-5) AML cells. Colonies with different signal intensity towards cDNA derived from resistant and sensitive AML were subjected to a second round of screening. DNA sequence reactions were carried out by the BigDye terminator method and analyzed by automatic sequencing (ABI PRISM). The BLAST search program was used for homology search (National Center for Biotechnology Information, Bethesda, MD).

### Dot-blot analysis of differentially expressed genes in cells from individual AML patients

Gene fragments from selected differentially expressed genes were amplified from plasmids by PCR using T7 promoter and M13 reverse primers. The amplified specific PCR fragments were used in dot-blot analysis using ^32^P-labeled cDNA probes from RNA of L1, 2, 3; R1, 2, 3, 4, 5 and H1, 2, 3, 4, and 5. The membranes were analyzed by phospho-imaging, and the hybridization signal normalized to the mRNA expression level of the ribosomal protein P2 (rpP2) gene for sample from each AML patient.

### 5'-RACE experiments

The 5'-end of ARAP mRNA was obtained by using 5'Generacer kit (Invitrogen) with primers described in the kit and antisense primers designed from the cDNA sequence. The antisense primers, 5'-GTCATCATCTTCTGAGTCTGACTC-3' and 5'-CACCATCATCACCTTGTTTTTGCC-3', were used for PCR amplification.

### Relative mRNA expression determined by RT-PCR

Total RNA from either NB4 cells or from patient AML blasts pooled from S1-3 or R1-5 was used for ss cDNA synthesis using oligo-(dT)-primer and superscript II reverse transcriptase (Invitrogen). The PCR reaction (50 μl) contained 1 μl cDNA and specific 5'-and 3'-primers. The PCR products were collected after 21–24 cycles of amplification. The primers were: rpL6, 5'-ATGGCGGGTGAAAAAGTTGAGAAGC-3' and 5'-GTAGAACACCAATTTGTGAGGA-3'; rpS4, 5'-ATGGCTCGTGGTCCCAAGAAGCATC-3' and 5'-GCACCCACTGCTCTGTTTGGCCGCC-3'; ARAP, 5'-CAAAAACAAGGTGATGATGATGG-3' and 5'-CCAGAGGGGCGGCAGCAGTCC-3'; Primers for full-length ARAP, 5'-ATGCCCAAATCCAAGCGCGACAA-3' and 5'-GTCATCATCTTCTGAGTCTGACTC-3'; LEDGF/p75, 5'-ATGACTCGCGATTTCAAACCTGGA-3' and 5'-GTTATCTAGTGTAGAATCCTTCAGA-3'; p52, 5'-ATGACTCGCGATTTCAAACCTGGA-3' and 5'-GTCCAATGAGTCTGTATCAAGATC-3'; p75-1, 5'-ATGACTCGCGATTTCAAACCTGGA-3' and 5'-GATCTTCATCTCTTGTTTGCTCCAC-3'; P75-2, 5'-AAGCCACCCACAAACAAACTACCC-3' and 5'-CCCTCCTTTTCTCTTCTTTTCTCC-3'; rpP2, 5'-ATGCGCTACGTCGCC-3' and 5'-TTAATCAAAAAGGCCAAATCCCAT-3'.

### Western blot analysis

Cell pellets were lyzed in ice-cold 10 mM Tris pH 7.5 with 0.5% NP-40, 1 mM MgCl_2_, 1 mM DTT, 10 mM KCl, 0.4 M NaCl for 5 min, and centrifuged at 11,000× g for 5 min. 50 μg supernatant protein was separated by 12% SDS-polyacrylamide gel electrophoresis under reducing conditions and transferred to a nitrocellulose filter. The membrane was probed with anti-LEDGF/p75 polyclonal antibodies (kindly provided by C. A. Casiano) and the membrane was developed using the enhanced ECL detection method (Amersham Biosciences, Uppsala, Sweden).

## Abbreviations

AML, acute myelogenous leukemia; ARAP, AML, resistance associated protein; LEDGF/P75, Lens Epithelial Derived Growth Factor; PWWP, proline-tryptophan-tryptophan-proline; ERF-2, EGF response factor 2; EF, Elongation Factor; MDS, myelodysplastic syndrome; PBMC, leukemic peripheral blood mononuclear cells

## Competing interests

The author(s) declare that they have no competing interests.

## Authors' contributions

TSH, LMM, EK and FP carried out most of the experiments. BTG and ØB provided all the cells from AML patients who were carefully diagnosed. BTG, SOD and JRL participated in designing and planning the experiments, and in writing the manuscript. All authors read and approved the final manuscript.
